# Enzyme Immobilization Strategies and Electropolymerization Conditions to Control Sensitivity and Selectivity Parameters of a Polymer-Enzyme Composite Glucose Biosensor

**DOI:** 10.3390/s100706439

**Published:** 2010-06-30

**Authors:** Sharon A. Rothwell, Sarah J. Killoran, Robert D. O’Neill

**Affiliations:** UCD School of Chemistry and Chemical Biology, University College Dublin, Belfield, Dublin 4, Ireland

**Keywords:** hydrogen peroxide, polyphenylenediamine, amperometry, enzyme-modified electrode, ascorbic acid interference, brain monitoring

## Abstract

In an ongoing programme to develop characterization strategies relevant to biosensors for *in-vivo* monitoring, glucose biosensors were fabricated by immobilizing the enzyme glucose oxidase (GOx) on 125 μm diameter Pt cylinder wire electrodes (Pt_C_), using three different methods: before, after or during the amperometric electrosynthesis of poly(*ortho*-phenylenediamine), PoPD, which also served as a permselective membrane. These electrodes were calibrated with H_2_O_2_ (the biosensor enzyme signal molecule), glucose, and the archetypal interference compound ascorbic acid (AA) to determine the relevant polymer permeabilities and the apparent Michaelis-Menten parameters for glucose. A number of selectivity parameters were used to identify the most successful design in terms of the balance between substrate sensitivity and interference blocking. For biosensors electrosynthesized in neutral buffer under the present conditions, entrapment of the GOx within the PoPD layer produced the design (Pt_C_/PoPD-GOx) with the highest linear sensitivity to glucose (5.0 ± 0.4 μA cm^−2^ mM^−1^), good linear range (*K*_M_ = 16 ± 2 mM) and response time (< 2 s), and the greatest AA blocking (99.8% for 1 mM AA). Further optimization showed that fabrication of Pt_C_/PoPD-GOx in the absence of added background electrolyte (*i.e.*, electropolymerization in unbuffered enzyme-monomer solution) enhanced glucose selectivity 3-fold for this one-pot fabrication protocol which provided AA-rejection levels at least equal to recent multi-step polymer bilayer biosensor designs. Interestingly, the presence of enzyme protein in the polymer layer had opposite effects on permselectivity for low and high concentrations of AA, emphasizing the value of studying the concentration dependence of interference effects which is rarely reported in the literature.

## 1. Introduction

The application of biosensors as analytical tools is a growing research topic in areas such as environmental surveillance, batch food analysis and clinical monitoring, and is beginning to impact on quality-of-life issues [1–5]. The choice of biosensor design for a particular application should be governed by diverse factors, including: the chemical nature of the analytical medium (e.g., lipophilic *versus* hydrophilic); the sample size (e.g., intracellular and extracellular monitoring *versus* batch analysis); the time resolution and recording duration required; and the concentration of the target analyte relative to the corresponding interference compounds for the chosen technique (electrochemical, optical, gravimetric, tonometric, thermal, magnetoelastic, *etc.*) [6–8]. For *in-vivo* monitoring in the brain during behavior, implantable biosensors showing good biocompatibility, sensitivity, selectivity and stability in this strongly lipophilic environment are needed, and amperometric enzyme-based devices incorporating a permselective polymer have been applied successfully in many neurochemical studies [9–17].

Poly-phenylenediamines (PPDs) electrosynthesized from one of the three monomer isomers have found widespread use as a biosensor permselectivity barrier [18–21], although poly(*ortho*-phenylenediamine), PoPD, may be superior for long-term *in-vivo* monitoring [22]. A variety of immobilization methods for oxidase enzymes (EOx) have also been described for PPD-based biosensors, with three approaches commonly used: enzyme deposited before the PPD layer, EOx/PoPD [23–26], enzyme immobilized over PPD, PPD/EOx [23,27–29] and enzyme co-immobilized from the monomer solution, PPD-EOx [30–32].

The amperometric enzyme-based biosensors used in this work were first generation devices which involve monitoring the formation of hydrogen peroxide, HP [33]. The first two reactions [Equations (1) and (2)] represent the enzyme (glucose oxidase, GOx) redox reactions, where FAD is the oxidized form of the prosthetic group, flavin adenine dinucleotide:

(1) $$\beta \text{-D-glucose}+\text{GOx}/\text{FAD}\to \text{D-glucono-}\delta \text{-lactone}+\text{GOx}/{\text{FADH}}_{2}$$

(2) $$\text{GOx}/{\text{FADH}}_{2}+O_{2}\to \text{GOx}/\text{FAD}+H_{2}O_{2}$$

(3) $$H_{2}O_{2}\to O_{2}+2H^{+}+2e^{-}$$

The H_2_O_2_ produced in Equation (2) can be oxidized, usually amperometrically, either directly on the electrode surface at relatively high applied potentials [Equation (3)] [33], or catalytically at lower potentials. However, even when significantly lower applied overpotentials can be used for H_2_O_2_ detection [34], interference by the ubiquitous biological reducing agent, ascorbic acid (AA), can persist because of its high concentration in most biological media and very low redox potential [35,36], and the use of redox-mediated HRP-based biosensors may suffer from indirect AA interference because HRP has been reported to catalyze the reaction between AA and H_2_O_2_ [37]. The incorporation of a permselective layer minimizes interference problems, and PoPD fulfils this function in many laboratories, blocking AA and other interference species well (dopamine, DOPAC, uric acid, *etc.*) while showing excellent permeability to H_2_O_2_ [10,11,18,25,26,31,38–40].

Recently, a number of new aspects to the problem of interference at PoPD-based biosensors have been identified. First, the permselectivity can be undermined for biosensors with large values of Pt-insulation “edge density”, such as microdisks [41]. Briefly, PoPD deposited near the electrode insulation is not as effective at blocking interference. Second, the incorporation of enzyme in the PoPD can decrease its blocking ability [41]. Third, electrosynthesis of enzyme-free PoPD in the absence of added background electrolyte can improve its permselective properties, apparently due to fewer ions being trapped in the polymer matrix [42]. Taking cognisance of these new findings, here we chose an implantable, low edge-density geometry (narrow Pt-Ir cylinders) as electrode substrate, and investigate the effects of different enzyme immobilization methods and electropolymerization conditions on the enzyme-kinetic and permeability parameters. Important aims of this study were to understand more fully factors affecting the characteristics of PoPD-based polymer-enzyme composite devices, and to determine whether optimizing the fabrication conditions of these single-polymer-layer biosensors could provide interference-rejection characteristics comparable to more complex sensing layers, such as those formed from multiple electrosynthesis and over-oxidation steps [43].

## 2. Experimental Section

### 2.1. Chemicals and Solutions

The enzyme glucose oxidase (GOx) from *Aspergillus niger* (180,200 U·g^−1^; EC 1.1.3.4, type VII-S) was obtained from Sigma-Aldrich, as were o*rtho-*phenylenediamine (oPD), α-d-( + )-glucose, ascorbic acid (AA), hydrogen peroxide (HP, 30% w/w aqueous solution) and potassium chloride. All reagents were used as supplied. All experimental calibrations were carried out in PBS (pH 7.4) prepared by adding NaCl (Sigma, 150 mM), NaH_2_PO_4_ (Fluka, 40 mM) and NaOH (Fluka, 40 mM) to distilled water, bubbled with N_2_ for 15 min, and stored at 4 °C. Solutions of monomer, oPD, were prepared in 25 mL of PBS, unless stated otherwise, and sonicated at room temperature until dissolved. A stock solution of 1 M glucose was prepared in distilled water and left for 24 h at room temperature to allow equilibration of the anomers, and then stored at 4 °C. Stock solutions of 10 mM HP and 100 mM AA were prepared in distilled water and 100 mM HCl, respectively.

### 2.2. Instrumentation and Software

Calibrations for HP, AA and glucose were performed in a standard three-electrode cell containing 20 mL PBS at room temperature, a saturated calomel reference electrode (SCE), a stainless steel auxiliary electrode and either bare or modified platinum-iridium (90:10) working electrodes. Constant potential amperometry was performed at an applied potential of +0.7 V *versus* SCE, using Chart (v 5.2) software (AD Instruments Ltd., Oxford, UK) and a low-noise potentiostat (Biostat IV, ACM Instruments, Cumbria, UK). The working electrodes were allowed to settle in quiescent PBS to give a steady background current before the addition of small known aliquots of the analyte of interest.

### 2.3. Working Electrode Preparation

**Figure 1.**
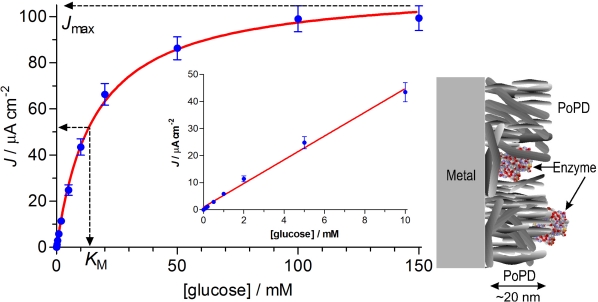
Sample steady-state calibration data and nonlinear regression analysis for the biosensor design, Pt_C_/PoPD-GOx [Equation (4), R^2^ = 0.998, n = 8; ***left***], illustrating the graphical significance of the Michaelis-Menten constants, *J*_max_ and *K*_M_. The linear region slope (LRS) was obtained using linear regression up to 10 mM glucose (R^2^ = 0.996, n = 8; ***left inset***), and represents the most suitable measure of analytical sensitivity of each biosensor design to enzyme substrate (see Table 1). Schematic representation of the PEC configuration for the same Pt_C_/PoPD-GOx design (***right***), illustrating trapped GOx (∼8 nm diameter) in the PoPD layer deposited by the precipitation of insoluble chains formed during the electropolymerization of monomer solution containing the enzyme.

Cylinder electrode preparation has been described in detail recently [44]. Briefly, 125 μm diameter Teflon-coated Pt-Ir wire (90:10, Advent Research Materials Ltd., Eynsham, England) was stripped of 1 mm Teflon to expose the bare metal, which displays many of the electrochemical properties of pure Pt [44]. Electropolymerization was carried out in oPD solutions (of varied monomer concentration, background electrolyte and enzyme concentration) at +0.7 V *versus* SCE for 15 minutes for these Pt_C_ electrodes [39,41]. Three main enzyme immobilization protocols were used in this work. In the first, the enzyme was immobilized by adsorption and dip-evaporation before PoPD deposition [23]. Each electrode was dipped in a 200 U·mL^−1^ solution of GOx for 5 minutes, allowed to dry for 5 min, and then dipped quickly into the GOx solution four more times with 5 minutes drying between each dip, followed by electropolymerization. This protocol was previously found to optimize enzyme loading for biosensors of the type Pt_C_/EOx/PoPD [24]. The second design immobilized the enzyme by adsorption and dip-evaporation after PoPD deposition followed by exposure to glutaraldehyde (GA) vapour for 15 min to crosslink the enzyme [23], and are termed Pt_C_/PoPD/GOx-GA. The third method used co-immobilization, whereby either 1 mg·mL^−1^ (∼650 U·mL^−1^; ∼5 μM) or 5 mg·mL^−1^ GOx was dissolved in oPD, and electropolymerized at +0.7 V *vs.* SCE for 15 min [30,31] to give Pt_C_/PoPD-GOx; see Figure 1.

### 2.4. Enzyme Kinetic Parameters

First generation biosensors of the general design Pt_C_/PoPD∼EOx (*i.e.*, various configurations of enzyme deposited before, over, or simultaneously with, the PoPD layer) display Michaelis-Menten kinetics, as discussed recently [11]. A previous study has shown that substrate diffusion is not limiting for non-conducting PoPD layers incorporating enzyme [32], due to their ultrathin nature (10–30 nm [31,45,46]). Therefore, the basic Michaelis-Menten enzyme parameters used here provide more readily accessible insights into factors affecting the responsiveness of biosensors fabricated from this polymer, and avoids the use of more complex analyses such as those involving the Thiele modulus [47].

Although a ping-pong mechanism describes the enzyme interaction with the substrate and co-substrate [Equations (1) and (2)], the oxygen effect was not included in the analysis here for simplicity. This is justified on two grounds: the concentration of oxygen was fixed in the present experiments (air saturation); and oxygen effects on biosensors of these designs are minimal for the range of substrate and oxygen levels encountered during neurochemical monitoring *in vivo* for both glucose [23,48] and glutamate [27,28]. Thus, the one-substrate form of the Michaelis-Menten equation contains the parameters used here to compare biosensor performance [Equation (4)], where *J*_S_ is the current-density normalized, background subtracted, biosensor response to a specified concentration of enzyme substrate ([S]):

(4) $$J_{s}=\frac{J_{\text{max}}}{1+K_{M}/[S]}$$

*J*_max_ is the maximum, or plateau, current density response, obtained when all enzyme sites are saturated with substrate (see Figure 1). Different values of *J*_max_, determined under the same conditions, reflect differences in the amount of active (not total) enzyme on the surface, provided *k*_cat_ and the sensitivity of the electrode to H_2_O_2_ [Equation (3)] does not vary much [11]. *K*_M_ is the apparent Michaelis constant, and phenomenologically defines the concentration of substrate that gives half the *J*_max_ response. Changes in *K*_M_ are sensitive to variations in enzyme-substrate access/binding, and have been interpreted in terms of barriers to enzyme-substrate access [26,49], as it is in the present study. *K*_M_ is also useful for defining the range of the linear response to S (up to ∼½*K*_M_), as well as determining the linear region slope (LRS), *i.e.*, LRS ≈ *J*_max_/*K*_M_ for a true hyperbolic response [11,27]. A plot of *J*_S_ *versus* [S] up to enzyme saturation therefore provides the basic kinetic parameters *J*_max_ and *K*_M_, as well as the nonlinear coefficient of determination, *R^2^*; a similar plot up to ∼½*K*_M_ provides the analytically key substrate sensitivity parameter, LRS, and the linear coefficient of determination, *R*^2^ (see Figure 1). To account for any variations in LRS caused by differences in polymer-enzyme composite (PEC) biosensor sensitivity to HP, the parameter BE% was defined as the HP-normalized LRS [Equation (5)], which can be considered to reflect the efficiency of the biosensor in converting substrate to HP [11]. This parameter also allows the efficiency of the enzyme layer to be compared across diverse biosensor designs:

(5) $$\text{BE}\%=\frac{\text{LRS at Pt}/\text{PEC}}{\text{slope}(\text{HP})\;\text{at Pt}/\text{PEC}}\times 100\%$$

### 2.5. Permeability and Permselectivity Parameters

From the calibration plots for HP, AA and glucose, a number of parameters were calculated to quantify the performance of the different designs. The apparent analyte permeabilities to HP and AA were calculated from Equations (6) and (7) [11], which is similar to other studies [50], and discussed in detail recently [41]:

(6) $$P(\text{HP})\%=\frac{\text{slope}(\text{HP})\;\text{at Pt}/\text{PEC}}{\text{slope}(\text{HP})\;\text{at bare Pt}}\times 100\%$$

**Figure 2.**
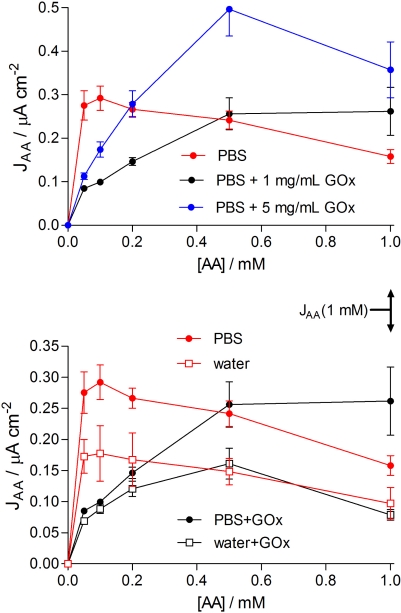
Averaged steady-state AA calibrations for Pt_C_/PoPD and Pt_C_/PoPD-GOx electrosynthesized in 300 mM oPD solution made with either PBS*, PBS + GOx (1 mg mL^−1^, *n* = 4), PBS+GOx (5 mg mL^−1^, *n* = 8), distilled water*, or water+GOx (1 mg mL^−1^, *n* = 6). The concentration of GOx for the bottom graph was 1 mg mL^−1^. *The GOx-free data were taken from the literature [38] for comparison.

The slopes (μA cm^−2^ mM^−1^) of the linear responses for HP on bare metal and on the PoPD-modified electrodes were obtained from linear regression analysis of the respective calibration plots of the steady-state HP responses *versus* HP concentration up to 0.1 mM, as were the AA slopes up to 1 mM for the bare electrodes. In contrast, the AA response was nonlinear and self limiting (see Figure 2), as observed previously for similar PoPD-based designs [30,41], and linked with “self-blocking” by AA-related species trapped in the polymer matrix [45]. Hence, the current density at 1 mM AA [*J*_AA_(1 mM)] was used as a measure of the AA response (see Figure 2). This AA concentration is relevant to neurochemical applications because baseline brain AA levels are ∼0.5 mM [51], reaching millimolar levels during periods of behavioral stimulation [52–54]. The ideal values of *P*(HP)% and *P*(AA)% for biosensor applications are therefore 100% and 0%, respectively. In addition, the concentration of other electroactive interference compounds in the brain, such as dopamine and its metabolites [55], are orders of magnitudes smaller than that of AA, ensuring that their contributions to PPD-based biosensor responses are insignificant [24,25,56–58]:

(7) $$P(\text{AA})\%=\frac{J_{\text{AA}}\left(1\;\text{mM}\right)\;\text{at Pt}/\text{PEC}}{J_{\text{AA}}\left(1\;\text{mM}\right)\;\text{at bare Pt}}\times 100\%$$

A polymer selectivity parameter, *S*% (Equation 8), has been defined as the percentage interference by AA in HP detection for equimolar concentrations [41,44], with an optimum value of 0%:

(8) $$S\%=\frac{J_{\text{AA}}\left(1\;\text{mM}\right)\;\text{at Pt}/\text{PEC}}{J_{\text{HP}}\left(1\;\text{mM}\right)\;\text{at Pt}/\text{PEC}}\times 100\%$$

Although *S*% has been a useful parameter for gaining insights into the performance of the enzyme-free electrosynthesized polymer [11], it is not a sufficient index of the selectivity of PEC-based biosensors. Hence *S*_S_%, the equimolar enzyme substrate selectivity parameter [Equation (9), e.g., *S*_G_% for glucose], has also been described [11], which is similar to, but more straightforward than, non-equimolar equivalents [50,59]. The ideal value of *S*_S_% is zero, and compares the capacity of the PEC layer to generate current from the enzyme reactions, *J*_S_ [see Equations (1–3)], to the interference response produced by AA, *J*_AA_, for 1 mM of each analyte, a concentration which is close to brain extracellular fluid values *in vivo* for both compounds [51,60]:

(9) $$S_{s}\%=\frac{J_{\text{AA}}\left(1\;\text{mM}\right)\;\text{at Pt}/\text{PEC}}{J_{s}\left(1\;\text{mM}\right)\;\text{at Pt}/\text{PEC}}\times 100\%$$

The permeability and selectivity parameters, as well as BE% [Equations (5–9)], reflect intrinsic properties of PoPD which are normalized with respect to actual electrochemical surface area, rather than geometrically calculated area. All parameters were determined for individual electrodes and then averaged over populations of sensors for each design. Results are reported are mean ± standard error (SEM), with *n* = number of electrodes. Currents are presented as current density, calculated using the geometric area of these smooth wire electrodes. Linear and nonlinear regression analyses were performed using GraphPad Prism (version 5.02, San Diego, CA, USA). The statistical significance of variations between parameters for the different designs was calculated using Student’s two-tailed unpaired *t*-tests (Prism 5.02), with values of *p* < 0.05 considered to indicate statistical significance of the difference.

## 3. Results and Discussion

A large body of work on GOx-based biosensors incorporating an electrosynthesized PPD permselective layer for glucose detection has been published over the past two decades, and a limited selection is cited here [10,11,18,20,26,31,32,38,39,46,61–63]. However, a broad range of variables are involved in the fabrication and characterization of these biosensors, including: which of the three phenylenediamine monomers is used; the concentration of monomer; the background electrolyte and pH of the electropolymerization medium; the choice of cyclic voltammetry or fixed applied potential (and the value of applied potential) in the PPD electrosynthesis step; the size and shape of the electrode substrate; the mode of enzyme immobilization and its concentration; the variety and concentration range of the interference species studied; flow *versus* quiescent calibration systems; surface imaging and spectrochemical characterization; *etc.* Therefore, further optimization of this system is possible, and would be useful both in terms of understanding the nature of the polymer-enzyme composite (PEC) layer (see Figure 1) and improving the performance of the biosensor device.

In our laboratory, significant enhancement of the glucose sensitivity for a PoPD-based biosensor has been described, by using a Pt disk geometry (Pt_D_) to increase GOx loading [23]. However, more recently, precise permeability data revealed a novel edge effect which compromised the ability of Pt_D_/PoPD devices to block interference [41]. Taken together, these reports highlight the need to integrate enzyme kinetic analysis and detailed polymer permselectivity in the characterization of specific biosensors [64]. The enzyme substrate selectivity parameter, *S*_S_% [Equation (9)], is a key measure of the balance between high enzyme sensitivity and low interference responses needed for practical biosensors. A number of literature studies have used a similar, non-equimolar, version of this parameter to good effect [39,50,59,65]. Here we apply an extensive range of enzyme kinetic and polymer permeability/permselectivity parameters, including *S*_S_%, to characterize and further optimize the properties of a low edge-density Pt_C_/PoPD-based biosensor electrosynthesized under different conditions, including the novel environment of no added background electrolyte [42].

### 3.1. Michaelis-Menten Characteristics of the Basic Designs

Three main methods of GOx immobilization were examined here: dip-evaporation before polymer electrosynthesis (Pt_C_/GOx/PoPD), dip-evaporation over the polymer using glutaraldehyde (GA) as a crosslinker (Pt_C_/PoPD/GOx-GA), and co-polymerization from the monomer solution (Pt_C_/PoPD-GOx). Glucose calibrations for biosensors of all designs followed Michaelis-Menten hyperbolic behavior [Equation (4), see Figure 1]; the corresponding *J*_max_, *K*_M_ and LRS values for biosensors fabricated using our standard background electrolyte and monomer concentration (PBS containing 300 mM oPD [24,30]) are given in Table 1, as well as parameters for the PoPD-free design, Pt_C_/GOx-GA, for comparison.

The PoPD-free design (Pt_C_/GOx-GA) displayed a moderate *J*_max_ value indicative of good active enzyme loading. The corresponding *K*_M_ value was the lowest of these six designs, indicating that the enzyme was readily accessible to the substrate, although values lower than 5 mM have been reported previously [66,67]. Addition of the PoPD layer after enzyme deposition (GOx/PoPD) decreased the *J*_max_ and increased the *K*_M_, the latter indicating that PoPD hindered access of the substrate to the surface-bound enzyme, as observed previously for a PoPD layer containing the protein, bovine serum albumin, BSA [23]. The decrease in *J*_max_ following deposition on the PoPD could be due to either covering of the GOx by the polymer, or displacement of the enzyme off the surface during the PoPD deposition. The finding that the *J*_max_ for Pt_C_/GOx-GA/PoPD was three-fold greater than that for Pt_C_/GOx/PoPD (see Table 1) suggests that, in the absence of crosslinking with GA, much of the enzyme is removed from the surface by electro-deposition of the polymer. This interpretation is consistent with the ultrathin nature of electrosynthesized PoPD [31,45,46] which is considered not to overwhelm immobilized enzyme [31,68]; see Figure 1.

Immobilization of GOx over the PoPD layer (PoPD/GOx) showed a 7-fold increase in active enzyme loading compared with the GOx/PoPD configuration, and was indistinguishable from the PoPD-free configuration (*p* > 0.34). Surprisingly, the *K*_M_ value was similar for PoPD/GOx and GOx/PoPD, suggesting that enzyme-substrate binding was compromised by the presence of PoPD (see Section 3.2). In an attempt to increase GOx loading further, GOx was dip-evaporated both before and after polymer fabrication (GOx/PoPD/GOx), with no benefit obtained (Table 1). Neither was any significant enhancement in *J*_max_ observed when a 2,000 U mL^−1^ GOx solution was used in the dip/evaporation procedure (data not shown).

In the final basic design, GOx (5 mg mL^−1^) was dissolved in the monomer solution for co-immobilization during electropolymerization, as described previously for other conditions and electrode geometries [30–32,50]. This PoPD-GOx configuration showed the highest active enzyme loading, nearly twice the value of the PoPD-free design (*p* < 0.001). The mean *K*_M_ value for Pt_C_/PoPD-GOx was not statistically different from the PoPD-free design (*p* > 0.33), indicating similar substrate-enzyme access. In addition, *K*_M_ for the PoPD-GOx configuration was significantly lower than for PoPD/GOx (*p* < 0.02). One speculation is that the different structure of PoPD deposited in the presence of solution GOx, which has been observed in scanning electron microscopy studies [30], is less obstructive to substrate binding, a notion supported by AA permeability data below (see Section 3.2). Irrespective of the mechanism, however, clearly the co-immobilization of GOx from the monomer solution is superior in terms of active enzyme loading and affinity (see Table 1) compared with these, and other BSA-containing, Pt_C_/PoPD∼GOx biosensor designs [23]. This detailed comparison supports the protocols advanced previously for the co-immobilization of enzyme with PoPD for biosensor fabrication [30–32,48,50,68].

The linear region slope (LRS) of the glucose calibration, a parameter determined by both the *J*_max_ and *K*_M_ values [11], is a better index of the functional sensitivity of the different designs. As expected, the deposition of GOx before the polymer led to the lowest LRS sensitivity, ∼8-fold lower than incorporation of enzyme over the polymer. This trend is in line with that reported recently, where BSA was incorporated in the PoPD matrix [23]. Co-polymerization of GOx displayed the highest LRS sensitivity, twice as good as the next ranking PoPD-based biosensor, Pt_C_/PoPD/GOx-GA (*p* < 0.003). LRS values are influenced by two main factors: the ability of the enzyme layer to convert substrate to HP (Equations (1)–(2)), and the sensitivity of the electrode to HP [Equation (3)]. This latter can be determined as the biosensor HP calibration slope, and normalization of LRS with respect to this HP slope provides an index of the efficiency of the biosensor to convert substrate to HP [BE%, Equation (5)]. As well as being of intrinsic interest, this parameter becomes of practical importance when biosensors are used in environments where HP is produced by other components in the medium, such as mitochondria in brain tissue [69]. The maximum value of BE% for the designs shown in Table 1 was ∼2% for the Pt_C_/GOx-GA and Pt_C_/PoPD-GOx configurations. This low value contrasts with estimates of ∼50% for PoPD-based glutamate biosensors, mainly due to the much higher affinity of glutamate oxidase for its substrate compared with the GOx-glucose system [70].

Response times were recorded in constantly stirred solution, using the PowerLab module operating at a data acquisition rate of > 100 Hz. A *t*_90%_ parameter was defined as the time taken for the analyte response to reach 90% of its maximum value from the start of the current upswing, and is similar to definitions used previously [71–73]. The co-immobilized PoPD-GOx design was used in this study because of its high enzyme loading and LRS sensitivity (Table 1). The response time for glucose was fast (*t*_90%_ = 1.7 ± 0.1 s, *n* = 6), with the corresponding response time for injections of HP aliquots (*t*_90%_ = 1.3 ± 0.1 s) indicating that only ∼0.4 s of the glucose response was attributable to the enzyme reactions [Equations (1–2)]. This compares favorably with a glutamate Pt_C_-based biosensor where glutamate oxidase was in the PoPD layer, and the glutamate component of the response time was ∼0.6 s [27]. These results are also consistent with previous time-response studies of the PoPD-GOx system [31], and with the ultrathin nature of PoPD, electrosynthesized under non highly-acidic conditions, allowing fast interaction of the enzyme with its substrate (see Figure 1). It appears, therefore, that *K*_M_ is a much more sensitive index of hindrance in enzyme-substrate interactions for these glucose biosensors (Table 1), as in the case of PoPD-based glutamate biosensors, where little difference in response time was observed across diverse PEC configurations with largely different *K*_M_ values [27].

### 3.2. Permeability Characteristics of the Basic Designs

Good permeability of the PEC membrane to HP is important for practical first-generation biosensor designs. The apparent HP permeability, *P*(HP)% defined by Equation (6), was similar for all designs (Table 2), with an average value of 106 ± 8% (*n* = 41) which was not significantly different from the ideal value of 100% (*p* > 0.42). Therefore differences in the permselectivity parameter, *S*% [Equation (8)], across the designs should be influenced mainly by polymer interference-rejection properties. Similarly, differences in the biosensor selectivity parameter, *S_S_*% [Equation (9)], should be due to a combination of polymer interference rejection and the ability of the PEC layer to generate HP. The finding that some *P*(HP)% values were greater than 100% is unexpected, but has been observed before for PoPD layers containing a variety of macromolecular modifiers [44]. These supra-optimal values may be due minor disproportionation of HP on metals, and its possible inhibition by polymer coatings [74].

The apparent AA permeability, *P*(AA)%, was calculated using Equation (7); all PoPD-modified designs blocked the 1 mM AA flux by ≥ 99% compared with the bare metal, with *P*(AA)% ≤ 1% (Table 2). The best blocking characteristics were displayed by Pt_C_/PoPD. *i.e.*, by the pure PoPD, with *P*(AA)% = 0.11 ± 0.02%, a value similar to that reported for PoPD deposited under the same conditions on pure, low edge-density, Pt microfiber electrodes [41]. The finding that *P*(AA)% for Pt_C_/PoPD/GOx (0.34 ± 0.05%, *p* < 0.001) was significantly greater than for the native polymer (Pt_C_/PoPD) suggests that the enzyme does not simply sit on top of the PoPD, but imbeds in the polymer, opening its structure and undermining its interference blocking to a small degree. Surprisingly, the GOx deposited before the PoPD had a similar effect on *P*(AA)% compared with the PoPD/GOx configuration. This view is consistent with the *K*_M_ data in Table 1, which shows that the barrier to GOx-glucose interactions was significantly greater for Pt_C_/PoPD/GOx compared with Pt_C_/GOx (*p* < 0.002), but not with Pt_C_/GOx/PoPD (*p* > 0.13). It is interesting to note that *P*(AA)% for the GOx/PoPD and PoPD/GOx configurations was additive when compared with Pt_C_/GOx/PoPD/GOx, the worst AA-rejecting configuration (1.0 ± 0.1%). This indicates that successive fabrication steps (in this case, GOx deposited both before and after the PoPD) perturb the polymer, compounding the slight undermining of its interference blocking properties. Finally, *P*(AA)% for the co-immobilized enzyme (GOx-PoPD, 0.24 ± 0.04%), which had the best glucose LRS value (Table 1), was second only to pure PoPD in terms of AA rejection (99.76%) for these basic designs.

To investigate whether the presence of GOx on the metal surface (GOx/PoPD design) or in the monomer solution (PoPD-GOx design) affected the rate of deposition of the PoPD, the collapse of electropolymerization current associated with the self-sealing nature of this polymer as it deposits on the electrode surface was analysed. The anodic electropolymerization current fell off very rapidly following the initial surge induced by the application of 0.7 V *versus* SCE, leading to a ∼99% loss of initial current by ∼10 s for electropolymerizations carried out in PBS containing 300 mM oPD. A 2-phase exponential decay model gave a significantly better nonlinear regression fit compared with a 1-phase analysis, as observed [22] and discussed [75] recently. The half-life values for the associated two time domains, *t*_½_(fast) and *t*_½_(slow), are a measure of the rate at which the blocking layer of PoPD builds up on the metal, and so might be expected to be influenced by the presence of protein macromolecules near the electrode surface. The reference values determined in the absence of GOx (*i.e.*, for Pt_C_/PoPD) were 60 ± 10 ms and 0.40 ± 0.02 s (*n* = 8), respectively. The presence of GOx on the surface prior to electro-deposition (Pt_C_/GOx/PoPD) did not significantly affect the rate of electropolymerization current decay: 50 ± 10 ms and 0.48 ± 0.06 s (*n* = 11, *p* > 0.28). In contrast, the present of GOx (5 mg mL^−1^) in the monomer solution did significantly slow the current collapse in both time domains: 140 ± 10 ms and 0.93 ± 0.06 s (*n* = 8, *p* < 0.001). Work is currently underway to understand more fully the significance of these fast and slower components of PoPD electrosynthesis [75], but here, as in recent findings [42], there does not appear to be any correlation between the rate of the electropolymerization current collapse and the apparent permeability of AA in the PoPD deposited (see Table 2 and Section 3.3).

As expected from the corresponding definitions and the relatively constant value of *P*(HP)% across the biosensor designs studied here (Table 2), the trend in the permselectivity parameter (*S*%), calculated using Equation (8), paralleled that of *P*(AA)% (see Table 2). It is the equimolar substrate selectivity (*S*_G_%), defined by Equation (9), which most clearly reveals the superiority of the co-immobilized configuration among these basic designs (Table 2). The mean *S*_G_% value for Pt_C_/PoPD-GOx (7 ± 1%, *n* = 8) was between 4 and 50 times smaller (better) than for the other designs. Thus, in media containing equal concentrations of glucose and AA at the ∼1-mM level, as is the case in brain extracellular fluid [51,60], the baseline response of the Pt_C_/PoPD-GOx biosensor would have a ∼7% interference contribution from AA. However, because *changes* in the biosensor signal are far more important than their absolute output, interference in monitoring glucose changes would be considerably less than this value due to the self-limiting shape of the AA response (see Figure 2).

### 3.3. Fine-Tuning the Conditions for Co-Immobilization of PoPD and GOx

Biosensors fabricated using the co-immobilization conditions described above show more than adequate substrate selectivity for glucose monitoring in most biological fluids. That said, it is always desirable to reduce biosensor interference as far as possible, especially in the design of biosensors of substrates which exist at much lower levels, such as when monitoring the key neurotransmitters, glutamate [76,77] and acetylcholine [78,79]. Electropolymerization conditions were therefore modified in attempts to lower further *S*_G_% for Pt_C_/PoPD-GOx devices compared with the standard conditions of 300 mM oPD in PBS containing 5 mg mL^−1^ GOx.

#### 3.3.1. Monomer Concentration

Previous studies have shown that there is little difference between the interference rejection properties of PoPD formed at widely different concentrations [24], and effective permselective PPD layers are often generated from solutions with monomer concentrations as low as 5 mM [19,31,46,46,80,81] and 3 mM [26,46,58]. However, for the detailed analysis and comparisons of the present study, 10 mM and 100 mM oPD were tested for the most selective basic design (Table 2), *i.e.*, co-immobilization of PoPD with 5 mg mL^−1^ GOx. In line with these cited reports, there was little difference between *P*(AA)% for all three oPD concentrations. Thus, although the highest concentration tested (300 mM, which is close to saturation) displayed the lowest (best) AA permeability (0.24 ± 0.04%, *n* = 8), there was no significant difference between this and *P*(AA)% determined for Pt_C_/PoPD-GOx biosensors electrosynthesized in 10 mM oPD (0.33 ± 0.05%, *n* = 4, *p* > 0.2).

However, large differences in the glucose Michaelis-Menten parameters were observed as a function of monomer concentration. Active enzyme loading was highest for 300 mM oPD (*J*_max_ = 111 ± 6 μA cm^−2^, *n* = 8; see Figure 1) and lowest for 10 mM (3.4 ± 0.5 μA cm^−2^, *n* = 4, *p* < 0.001). The enzyme affinity was also greatest for 300 mM monomer (*K*_M_ = 16 ± 2 mM, n = 8) compared with 10 mM oPD (*K*_M_ = 36 ± 3 mM, *n* = 4, *p* < 0.001). These data are consistent with the notion that such a low population density of GOx molecules in the PEC layer would be more hindered by the polymer, as observed previously in a detailed analysis of the correlation between GOx loading and *K*_M_ for Pt_C_-based glucose biosensors [23]. These two factors (enzyme loading and affinity) combined to produced a 50-fold decrease in the LRS for biosensors fabricated in 10 mM oPD (LRS = 0.09 ± 0.02 μA cm^−2^ mM^−1^, *n* = 4) compared with 300 mM monomer (5.0 ± 0.4 μA cm^−2^ mM^−1^, *n* = 8, *p* < 0.001), which is the opposite trend reported for PoPD-based glucose biosensors made in different oPD concentrations, using an FIA system [50]. The oPD concentration was therefore maintained at 300 mM throughout the remainder of this investigation.

#### 3.3.2. Enzyme Concentration

The polymerization conditions for the basic co-immobilization design (Figure 1, and Tables 1 and 2) involved 5 mg mL^−1^ (∼3 kU mL^−1^) GOx in the monomer solution because this enzyme concentration had been adopted in previous studies to optimise selectivity for disk-based biosensors fabricated in PBS [30]. Given our greater understanding now of the different factors affecting the performance characteristics of disk *versus* cylinder biosensors [23,27,28,41,70], and the more common use of lower enzyme activity solutions for co-immobilization of GOx [31,50,82,83], Pt_C_/PoPD-GOx biosensors made from 1 mg mL^−1^ GOx (∼650 U mL^−1^; ∼5 μM) in PBS containing 300 mM oPD were characterized (see Table 3). There was only a small, but statistically significant, decrease in glucose calibration mean *J*_max_ values for biosensors fabricated in 1 mg mL^−1^ *versus* 5 mg mL^−1^ GOx solutions (*p* < 0.04), with no significant difference between the *K*_M_ values (*p* > 0.4). Not surprisingly, therefore, both the mean LRS and BE% values were indistinguishable for the two populations of biosensors (*p* > 0.9). Moreover, the finding that the 1-mM AA rejection parameter *P*(AA)% was not significantly different (*p* > 0.3; see Figure 2) meant that *S*% and *S*_G_% were also indistinguishable for the two groups (Table 3).

There were, however, subtle effects of different enzyme concentrations in the polymerization solution on the subsequent AA calibration responses (see Figure 2, top). When no protein was present, the maximum steady-state AA current was observed at low AA levels (∼0.1 mM), and the response diminished gradually and steadily thereafter. When 1 mg mL^−1^ GOx was included in the polymerization medium (*i.e.*, for Pt_C_/PoPD-GOx_1_), the AA calibration was more hyperbolic with significantly smaller AA currents at lower AA concentrations. The higher concentration of enzyme (5 mg mL^−1^) in the monomer solution (Pt_C_/PoPD-GOx_5_), however, led to greater responses for all AA concentrations compared with the 1 mg mL^−1^ level (Figure 2, top). Therefore, because the additional enzyme activity in the polymerization medium did not increase substrate sensitivity for the resulting biosensors, and because the shape of the AA calibrations recorded with Pt_C_/PoPD-GOx_1_ were more benign for applications involving AA-containing biological media, the 1 mg mL^−1^ GOx concentration in 300 mM oPD was used throughout the remainder of this work.

#### 3.3.3. Electropolymerization Background Electrolyte

A recent study showed that omission of added background electrolyte from the oPD (weak electrolyte) solution slowed down the electropolymerization current collapse by two orders of magnitude, but surprisingly augmented the blocking ability of the enzyme-free Pt_C_/PoPD formed [42]. Biosensors were therefore fabricated here in solutions of 1 mg mL^−1^ GOx dissolved in distilled water containing 300 mM oPD, and characterized in terms of Michaelis-Menten, permeability and selectivity parameters (see Table 3). The collapse in the electropolymerization current was slow in the absence of added background electrolyte for Pt_C_/PoPD(H_2_O), with a 1-phase exponential decay half-life value of 228 ± 20 s, *n* = 9. Incorporation of 1 mg mL^−1^ GOx in the 300 mM oPD distilled water solution marginally accelerated the formation of the self-sealing polymer layer (half life value of 159 ± 12 s, *n* = 3, *p* < 0.09), reflecting the increase in solution conductivity caused by the presence of the protein polyelectrolyte.

There was no significant difference between any of the enzyme parameters, including BE%, for biosensors generated in PBS compared with no added background electrolyte (Table 3). In line with previous enzyme-free studies [42], the mean *P*(AA)% for Pt_C_/PoPD-GOx(H_2_O) was ∼40% less than that determined for Pt_C_/PoPD-GOx(PBS). There was also no significant difference (*p* > 0.8) between *P*(AA)% determined for Pt_C_/PoPD(H_2_O) *versus* Pt_C_/PoPD-GOx_1_(H_2_O), indicating that the ∼5 μM level of polyelectrolyte enzyme was low enough not to affect the structure of the PoPD in such a way as to influence this parameter. In addition to this improvement in AA blocking at 1 mM levels, there was a decrease in the AA response across the entire concentration range of the AA calibration (see Figure 2, bottom). In view of these observations and the finding that AA responses at low AA levels were significantly decreased by this concentration of GOx in the polymerization solution for both PBS and no background electrolyte conditions (Figure 2), there is clearly much remaining to be understood about the structure of surface PoPD electro-deposited in non-acidic media [30,84–86], the influence of trapped enzymes, and details of the interactions of the PEC layer with AA during calibrations. At the analytically useful phenomenological level, however, there was a ∼3-fold improvement in *S*_G_% for biosensors fabricated in the absence of background electrolyte (Table 3).

**Figure 3.**
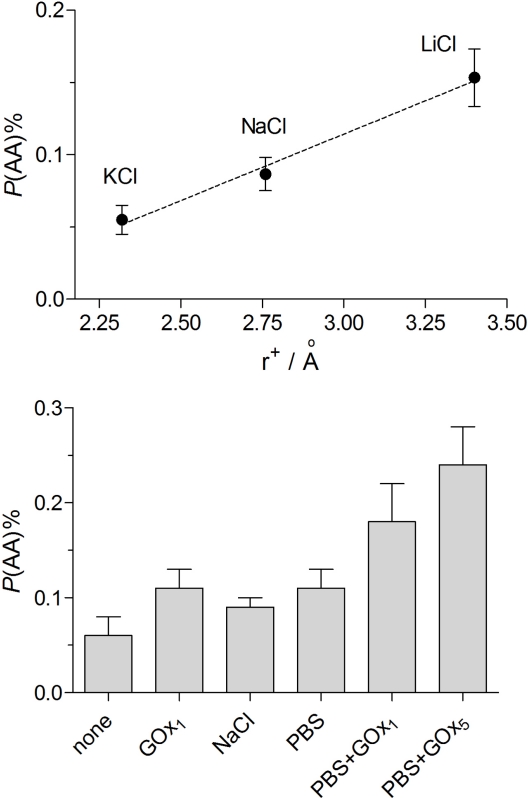
Effect of different background electrolytes and GOx concentrations in the monomer solution on the subsequent *P*(AA)% values determined for Pt_C_/PoPD electrodes electrosynthesized from 300 mM oPD. ***Top:*** 150 mM of either KCl (*n* = 12), NaCl (*n* = 8) or LiCl (*n* = 4) plotted against the hydrodynamic radius of the cations. ***Bottom*** (left to right): no added background electrolyte (*i.e.*, distilled water, *n* = 28); distilled water containing 1 mg mL^−1^ GOx (*n* = 6), 150 mM NaCl (*n* = 8); phosphate buffered 150 mM NaCl (PBS, *n* = 19); PBS containing 1 mg mL^−1^ GOx (*n* = 4); or PBS containing 5 mg mL^−1^ GOx (*n* = 8).

In view of these, and published [42,87], findings that the composition of the background electrolyte affects the permeability of even the non-conducting, non-ionic form of this polymer electrogenerated at non-acidic pH [88], the effects of different alkali metal salts in the monomer solution were investigated in the first instance for the enzyme-free Pt_C_/PoPD devices. Figure 3 (top) shows the trend in *P*(AA)% for these three electrolytes at 150 mM levels in the polymerization solution. There was a consistent decrease (improvement) in AA permeability with decreasing hydrated cation radius, with the optimum value of *P*(AA)% achieved for KCl solutions (0.05 ± 0.01%, *n* = 12), a value indistinguishable from the no-added-electrolyte condition (0.07 ± 0.02%, *n* = 28, *p* > 0.5). It is interesting to note that the precision of these apparent permeability measurements was sufficient to reveal this subtle trend in the influence of alkali-metal cation hydrodynamic radius on *P*(AA)%.

Because of the logistic advantages of faster electropolymerization times, biosensors made using 1 mg mL^−1^ GOx in 150 mM KCl containing 300 mM oPD were characterized (see Table 3). Whereas enzyme loading was similar to the other designs in Table 3, the *K*_M_ value was unexpectedly high, which led to a poor LRS value. Thus, although the *P*(AA)% for this biosensor configuration was as good as the no-added-electrolyte condition, the *S*_G_% value was poor because of lower substrate sensitivity. It appears, therefore, that 1 mg mL^−1^ GOx in 300 mM oPD dissolved in distilled water provided the best overall combination of good glucose sensitivity and interference (AA) rejection, yielding a biosensor with a *S*_G_% value of 2 ± 1%.

Finally, Figure 3 (bottom) shows the trend in *P*(AA)% for PoPD-modified electrodes electrosynthesized in 300 mM oPD solutions for the key conditions explored in this study. The lowest (best) value was observed for the non-biosensing device prepared from the monomer dissolved in distilled water only. Addition of 1 mg mL^−1^ GOx (∼5 μM) to the oPD solution doubled the subsequently determined mean *P*(AA)% value, whereas addition of 150 mM NaCl produced a slightly smaller detrimental effect than the much lower concentration of the macromolecular polyanion. Further addition of 40 mM phosphate ions to the 150 mM NaCl (*i.e.*, PBS) increased *P*(AA)% only slightly and insignificantly. The inclusion of 1 and 5 mg mL^−1^ GOx in the PBS-based oPD solution progressively increased *P*(AA)%. However, the total increase in AA permeability was only a factor of four across all these conditions, so that the pure PoPD polymer (Pt_C_/PoPD_water_) blocked 1 mM AA by 99.94 ± 0.02% while the worst blocking was displayed by Pt_C_/PoPD_PBS_-GOx_5_ (99.76 ± 0.04%).

The value of *P*(AA)% for the optimized biosensor, Pt_C_/PoPD-GOx_1_(H_2_O), was as low as 0.11 ± 0.02% (*n* = 6; see Table 3 and Figure 3). Many literature biosensor characterization studies do not report *P*(AA)%, or equivalent values. However, a recent paper did report AA currents for bare and polymer-coated electrodes incorporating a novel electrosynthesized polymeric bilayer membrane composed of overoxidized poly(pyrrole) and poly(2-naphthol) films [43]. These data allow an approximate *P*(AA)% equivalent to be calculated: 0.13 ± 0.02%, which is not superior to the single-pot fabrication described here for Pt_C_/PoPD-GOx_1_(H_2_O), and highlight the outstanding permselective properties of PoPD electrosynthesized under the present optimized conditions.

## 4. Conclusions

The analyses and results presented here demonstrate that precise measurement of PEC permeability characteristics can reveal subtle variations in the behavior of the polymer-enzyme composite layer which have important implications for biosensor design. Although variations on a common theme, all the PoPD-based biosensor designs in the present study are novel in their detail. The optimum biosensor for glucose was achieved by co-immobilizing 1 mg mL^−1^ GOx in 300 mM oPD dissolved in distilled water, a condition not reported for biosensor fabrication to date. This design showed a 3-fold superior substrate selectivity with respect to AA compared with the standard electropolymerization medium which has heretofore included an added background electrolyte, usually buffered close to neutrality. The influence of minor deviations from neutrality in these non-buffered monomer (weak base electrolyte) solutions, as well as ion-size factors, have been discussed previously for enzyme-free PoPD [42].

The improvements reported here are useful, but not critical, for glucose monitoring because of its high concentration in many body fluids. However, the approach described will help develop useful strategies in the design of biosensors for biological substrates which exist at much lower levels, such as when monitoring the key neurotransmitters, glutamate [76,77] and acetylcholine [43,78,79]. For example, the presence of enzyme protein in the polymer layer had opposite effects on permselectivity for low and high concentrations of AA, emphasizing the value of studying the concentration dependence of interference effects which is rarely reported in the literature.

Further strategies are available, such as the platinization of the smooth wire surface before PEC modification, which can increase the LRS by 60-fold and enhance BE% for a PoPD-GOx layer to ∼10% [32]. A cost-benefit analysis would need to be carried out, however, before the incorporation of a further step in the fabrication process, the result of which would depend on the concentrations of the analyte and interference species in the target medium. Overall, this latest optimization of glucose biosensors based on a PoPD permselective layer, demonstrates that the one-pot fabrication of Pt_C_/PoPD-GOx in the absence of added background electrolyte provides a device with AA-rejection characteristics comparable to more complex sensing layers, such as those formed from multiple electrosynthesis and over-oxidation steps [43].

## Figures and Tables

**Table 1.** Mean ± SEM (*n* = number of electrodes) for the two apparent Michaelis-Menten parameters *J*_max_ and *K*_M_ determined using nonlinear regression and Equation (4) for glucose calibrations (see Figure 1), and the corresponding linear region slope (LRS) values. PoPD-based biosensors were electrosynthesized in PBS containing 300 mM oPD and no enzyme, except for the Pt_C_/PoPD-GOx design which included 5 mg mL^−1^ GOx. The PoPD-free design is included for comparison.

**Design**	***n*-value**	***J*_max_ (μA cm^−2^)**	***K*_M_ (mM)**	**LRS (μA cm^−2^ mM^−1^)**	**BE% (%)**
Pt_C_/GOx-GA	4	66 ± 5	13 ± 1	3.6 ± 0.2	1.4 ± 0.1
Pt_C_/GOx-GA/PoPD	4	33 ± 3	32 ± 3	1.2 ± 0.1	0.47 ± 0.05
Pt_C_/GOx/PoPD	7	10 ± 1	21 ± 2	0.37 ± 0.03	0.17 ± 0.01
Pt_C_/PoPD/GOx-GA	4	72 ± 3	26 ± 2	2.7 ± 0.1	0.66 ± 0.02
Pt_C_/GOx/PoPD/GOx-GA	4	50 ± 5	26 ± 3	1.7 ± 0.4	0.55 ± 0.07
Pt_C_/PoPD-GOx	8	111 ± 6	16 ± 2	5.0 ± 0.4	2.0 ± 0.2

**Table 2.** Mean values ± SEM (*n* = number of electrodes) for the two apparent permeabilities, *P*(HP)% and *P*(AA)% determined using Equations (6) and (7), respectively, and for the two selectivity parameters, *S*% and *S*_G_%, defined by Equations (8) and (9), respectively, for both PEC-coated and enzyme-free PoPD-modified Pt_C_ electrodes. The electropolymerization solution contained 300 mM oPD in PBS, and 5 mg mL^−1^ GOx for the co-immobilization design (Pt_C_/PoPD-GOx).

**Design**	***n*-value**	***P*(HP)%**	***P*(AA)%**	**S%**	***S*_G_%**
Pt_C_/PoPD	19	90 ± 5	0.11 ± 0.02	0.09 ± 0.01	N/A*
Pt_C_/GOx/PoPD	6	97 ± 7	0.34 ± 0.05	0.20 ± 0.03	139 ± 36
Pt_C_/PoPD/GOx-GA	4	98 ± 2	0.51 ± 0.07	0.29 ± 0.03	29 ± 8
Pt_C_/GOx/PoPD/GOx-GA	4	127 ± 8	1.0 ± 0.1	0.55 ± 0.07	108 ± 32
Pt_C_/PPD-GOx	8	120 ± 7	0.24 ± 0.04	0.14 ± 0.02	7 ± 1

* Not applicable, because enzyme-free designs do not respond to glucose.

**Table 3.** Mean values ± SEM for the apparent Michaelis-Menten parameters *J*_max_ and *K*_M_ determined using nonlinear regression [Equation (4)] for glucose calibrations and the linear region slope (LRS) for Pt_C_/PoPD-GOx biosensors electrosynthesized in different media. The apparent AA permeability *P*(AA)% determined using Equation (7) and the two selectivity parameters, *S*% and *S*_G_%, determined using Equations (8) and (9), respectively, for these biosensors fabricated in 300 mM oPD and 1 mg mL^−1^ GOx (unless stated otherwise) dissolved in different added background electrolytes: PBS (5 mg mL^−1^ GOx, *n* = 8), PBS (*n* = 4), KCl (*n* = 4) and no added background electrolyte (distilled water, n = 6).

**Background electrolyte:**	**PBS**	**PBS**	**KCl**	**none**
**[GOx] (mg mL^−1^):**	5.0	1.0	1.0	1.0
***J*_max_ (μA cm^−2^)**	111 ± 6	85 ± 7	87 ± 3	96 ± 5
***K*_M_ (mM)**	16 ± 2	13 ± 2	27 ± 1	10 ± 1
**LRS (μA cm^−2^ mM^−1^)**	5.0 ± 0.4	5.0 ± 1.0	3.1 ± 0.2	6.5 ± 0.6
***BE%***	2.0 ± 0.2	2.0 ± 0.4	1.1 ± 0.1	2.4 ± 0.2
***P*(AA)%**	0.24 ± 0.04	0.18 ± 0.04	0.11 ± 0.01	0.11 ± 0.02
**S%**	0.14 ± 0.02	0.09 ± 0.02	0.06 ± 0.01	0.07 ± 0.01
***S*_G_%**	7 ± 1	7 ± 2	6 ± 1	2 ± 1

## References

[1] Hirst ER, Yuan YJ, Xu WL, Bronlund JE (2008). Bond-rupture immunosensors—A review. Biosens. Bioelectron.

[2] Wanekaya AK, Chen W, Mulchandani A (2008). Recent biosensing developments in environmental security. J. Environ. Monit.

[3] Sadik OA, Aluoch AO, Zhou AL (2009). Status of biomolecular recognition using electrochemical techniques. Biosens. Bioelectron.

[4] Sozer N, Kokini JL (2009). Nanotechnology and its applications in the food sector. Trends Biotechnol.

[5] Saito H, Nakazato T, Ishii N, Kudo H, Otsuka K, Endo H, Mitsubayashi K (2008). An optical flow injection analysis system for measurement of glucose in tomato. Eur. Food Res. Technol.

[6] Wei D, Bailey MJA, Andrew P, Ryhanen T (2009). Electrochemical biosensors at the nanoscale. Lab Chip.

[7] Mitsubayashi K, Ohgoshi T, Okamoto T, Wakabayashi Y, Kozuka M, Miyajima K, Saito H, Kudo H (2009). Tonometric biosensor with a differential pressure sensor for chemo-mechanical measurement of glucose. Biosens. Bioelectron.

[8] Lu QZ, Lin HL, Ge ST, Luo SL, Cai QY, Grimes CA (2009). Wireless, remote-query, and high sensitivity escherichia coli O157:H7 biosensor based on the recognition action of Concanavalin A. Anal. Chem.

[9] Tian FM, Gourine AV, Huckstepp RTR, Dale N (2009). A microelectrode biosensor for real time monitoring of L-glutamate release. Anal. Chim. Acta.

[10] Calia G, Rocchitta G, Migheli R, Puggioni GM, Spissu Y, Bazzu G, Mazzarello V, Lowry JP, O’Neill RD, Desole MS, Serra PA (2009). Biotelemetric monitoring of brain neurochemistry in conscious rats, using microsensors and biosensors. Sensors.

[11] O’Neill RD, Lowry JP, Rocchitta G, McMahon CP, Serra PA (2008). Designing sensitive and selective polymer/enzyme composite biosensors for brain monitoring *in vivo*. Trends Anal. Chem.

[12] Pernot P, Mothet JP, Schuvailo O, Soldatkin A, Pollegioni L, Pilone M, Adeline MT, Cespuglio R, Marinesco S (2008). Characterization of a yeast D-amino acid oxidase microbiosensor for D-serine detection in the central nervous system. Anal. Chem.

[13] Wang J (2008). Electrochemical glucose biosensors. Chem. Rev.

[14] Wilson GS, Gifford R (2005). Biosensors for real-time *in vivo* measurements. Biosens. Bioelectron.

[15] Burmeister JJ, Gerhardt GA (2003). Ceramic-based multisite microelectrode arrays for *in vivo* electrochemical recordings of glutamate and other neurochemicals. Trends Anal. Chem.

[16] Pantano P, Kuhr WG (1995). Enzyme-modified microelectrodes for *in vivo* neurochemical measurements. Electroanalysis.

[17] Lowry JP, Ryan MR, O’Neill RD (1998). Behaviourally induced changes in extracellular levels of brain glutamate monitored at 1 s resolution with an implanted biosensor. Anal. Commun.

[18] Schuvailo OM, Soldatkin OO, Lefebvre A, Cespuglio R, Soldatkin AP (2006). Highly selective microbiosensors for *in vivo* measurement of glucose, lactate and glutamate. Anal. Chim. Acta.

[19] Dai YQ, Zhou DM, Shiu KK (2006). Permeability and permselectivity of polyphenylenediamine films synthesized at a palladium disk electrode. Electrochim. Acta.

[20] Carelli I, Chiarotto I, Curulli A, Palleschi G (1996). Electropolymerization of hydroxybenzene and aminobenzene isomers on platinum electrodes to assemble interference-free electrochemical biosensors. Electrochim. Acta.

[21] Li XG, Huang MR, Duan W, Yang YL (2002). Novel multifunctional polymers from aromatic diamines by oxidative polymerisations. Chem. Rev.

[22] Killoran SJ, O’Neill RD (2008). Characterization of permselective coatings electrosynthesized on Pt-Ir from the three phenylenediamine isomers for biosensor applications. Electrochim. Acta.

[23] McMahon CP, Killoran SJ, O’Neill RD (2005). Design variations of a polymer-enzyme composite biosensor for glucose: Enhanced analyte sensitivity without increased oxygen dependence. J. Electroanal. Chem.

[24] Ryan MR, Lowry JP, O’Neill RD (1997). Biosensor for neurotransmitter L-glutamic acid designed for efficient use of L-glutamate oxidase and effective rejection of interference. Analyst.

[25] Cooper JM, Foreman PL, Glidle A, Ling TW, Pritchard DJ (1995). Glutamate oxidase enzyme electrodes: microsensors for neurotransmitter determination using electrochemically polymerized permselective films. J. Electroanal. Chem.

[26] Sasso SV, Pierce RJ, Walla R, Yacynych AM (1990). Electropolymerized 1, 2-diaminobenzene as a means to prevent interferences and fouling and to stabilize immobilized enzyme in electrochemical biosensors. Anal. Chem.

[27] McMahon CP, Rocchitta G, Serra PA, Kirwan SM, Lowry JP, O’Neill RD (2006). Control of the oxygen dependence of an implantable polymer/enzyme composite biosensor for glutamate. Anal. Chem.

[28] McMahon CP, Rocchitta G, Kirwan SM, Killoran SJ, Serra PA, Lowry JP, O’Neill RD (2007). Oxygen tolerance of an implantable polymer/enzyme composite glutamate biosensor displaying polycation-enhanced substrate sensitivity. Biosens. Bioelectron.

[29] O’Brien KB, Killoran SJ, O’Neill RD, Lowry JP (2007). Development and characterization *in vitro* of a catalase-based biosensor for hydrogen peroxide monitoring. Biosens. Bioelectron.

[30] Lowry JP, O’Neill RD (1994). Partial characterization *in vitro* of glucose oxidase-modified poly(phenylenediamine)-coated electrodes for neurochemical analysis *in vivo*. Electroanalysis.

[31] Malitesta C, Palmisano F, Torsi L, Zambonin PG (1990). Glucose fast-response amperometric sensor based on glucose oxidase immobilized in an electropolymerized poly(o-phenylenediamine) film. Anal. Chem.

[32] Reyes De Corcuera JI, Cavalieri RP, Powers JR (2005). Improved platinization conditions produce a 60-fold increase in sensitivity of amperometric biosensors using glucose oxidase immobilized in poly-o-phenylenediamine. J. Electroanal. Chem.

[33] Guilbault GG (1984). Analytical Uses of Immobilised Enzymes.

[34] Lee CH, Wang SC, Yuan CJ, Wen MF, Chang KS (2007). Comparison of amperometric biosensors fabricated by palladium sputtering, palladium electrodeposition and Nafion/carbon nanotube casting on screen-printed carbon electrodes. Biosens. Bioelectron.

[35] Kulys J, Drungiliene A (1991). Electrocatalytic oxidation of ascorbic acid at chemically modified electrodes. Electroanalysis.

[36] El Atrash SS, O’Neill RD (1995). Characterisation *in vitro* of a naphthoquinone-mediated glucose oxidase-modified carbon paste electrode designed for neurochemical analysis *in vivo*. Electrochim. Acta.

[37] Lin YQ, Liu K, Yu P, Xiang L, Li XC, Mao LQ (2007). A facille electrochemical method for simultaneous and on-line measurements of glucose and lactate in brain microdialysate with prussian blue as the electrocatalyst for reduction of hydrogen peroxide. Anal. Chem.

[38] Fu YC, Chen C, Xie QJ, Xu XH, Zou C, Zhou QM, Tan L, Tang H, Zhang YY, Yao SZ (2008). Immobilization of enzymes through one-pot chemical preoxidation and electropolymerization of dithiols in enzyme-containing aqueous suspensions to develop biosensors with improved performance. Anal. Chem.

[39] Lowry JP, McAteer K, El Atrash SS, Duff A, O’Neill RD (1994). Characterization of glucose oxidase-modified poly(phenylenediamine)-coated electrodes *in vitro* and *in vivo*: Homogeneous interference by ascorbic acid in hydrogen peroxide detection. Anal. Chem.

[40] Wang J, Chen L, Liu J, Lu F (1996). Enhanced selectivity and sensitivity of first-generation enzyme electrodes based on the coupling of rhodinized carbon paste transducers and permselective poly(o-phenylenediamine) coatings. Electroanalysis.

[41] Rothwell SA, Kinsella ME, Zain ZM, Serra PA, Rocchitta G, Lowry JP, O’Neill RD (2009). Contributions by a novel edge effect to the permselectivity of an electrosynthesized polymer for microbiosensor applications. Anal. Chem.

[42] Rothwell SA, Killoran SJ, Neville EM, Crotty AM, O’Neill RD (2008). Poly(o-phenylenediamine) electrosynthesized in the absence of added background electrolyte provides a new permselectivity benchmark for biosensor applications. Electrochem. Commun.

[43] Guerrieri A, Lattanzio V, Palmisano F, Zambonin PG (2006). Electrosynthesized poly(pyrrole)/poly(2-naphthol) bilayer membrane as an effective anti-interference layer for simultaneous determination of acethylcholine and choline by a dual electrode amperometric biosensor. Biosens. Bioelectron.

[44] Kirwan SM, Rocchitta G, McMahon CP, Craig JD, Killoran SJ, O’Brien KB, Serra PA, Lowry JP, O’Neill RD (2007). Modifications of poly(*o*-phenylenediamine) permselective layer on Pt-Ir for biosensor application in neurochemical monitoring. Sensors.

[45] Craig JD, O’Neill RD (2003). Comparison of simple aromatic amines for electrosynthesis of permselective polymers in biosensor fabrication. Analyst.

[46] Myler S, Eaton S, Higson SPJ (1997). Poly(o-phenylenediamine) ultra-thin polymer-film composite membranes for enzyme electrodes. Anal. Chim. Acta.

[47] Gooding JJ, Hall EAH (1996). Parameters in the design of oxygen detecting oxidase enzyme electrodes. Electroanalysis.

[48] Dixon BM, Lowry JP, O’Neill RD (2002). Characterization *in vitro* and *in vivo* of the oxygen dependence of an enzyme/polymer biosensor for monitoring brain glucose. J. Neurosci. Meth.

[49] Compagnone D, Federici G, Bannister JV (1996). A new conducting polymer glucose sensor based on polythianaphthene. Electroanalysis.

[50] Centonze D, Guerrieri A, Malitesta C, Palmisano F, Zambonin PG (1992). An in-situ electrosynthesized poly-ortho-phenylenediamine/glucose oxidase amperometric biosensor for flow-injection determination of glucose in serum. Ann. Chim. (Rome).

[51] Miele M, Fillenz M (1996). *In vivo* determination of extracellular brain ascorbate. J. Neurosci. Meth.

[52] Boutelle MG, Svensson L, Fillenz M (1989). Rapid changes in striatal ascorbate in response to tail-pinch monitored by constant potential voltammetry. Neuroscience.

[53] O’Neill RD, Fillenz M, Albery WJ (1983). The development of linear sweep voltammetry with carbon paste electrodes *in vivo*. J. Neurosci. Meth.

[54] Fillenz M, O’Neill RD (1986). Effects of light reversal on the circadian pattern of motor activity and voltammetric signals recorded in rat forebrain. J. Physiol. (London).

[55] Brose N, O’Neill RD, Boutelle MG, Anderson SMP, Fillenz M (1987). Effects of an anxiogenic benzodiazepine receptor ligand on rat motor activity and dopamine release in nucleus accumbens and striatum. J. Neurosci.

[56] Soldatkin OO, Schuvailo OM, Marinesco S, Cespuglio R, Soldatkin AR (2009). Microbiosensor based on glucose oxidase and hexokinase co-immobilised on platinum microelectrode for selective ATP detection. Talanta.

[57] Santos RM, Lourenco CF, Piedade AP, Andrews R, Pomerleau F, Huettl P, Gerhardt GA, Laranjinha J, Barbosa RM (2008). A comparative study of carbon fiber-based microelectrodes for the measurement of nitric oxide in brain tissue. Biosens. Bioelectron.

[58] Hamdi N, Wang JJ, Monbouquette HG (2005). Polymer films as permselective coatings for H_2_O_2_-sensing electrodes. J. Electroanal. Chem.

[59] McAteer K, O’Neill RD (1996). Strategies for decreasing ascorbate interference at glucose oxidase-modified poly(o-phenylenediamine)-coated electrodes. Analyst.

[60] Lowry JP, O’Neill RD, Boutelle MG, Fillenz M (1998). Continuous monitoring of extracellular glucose concentrations in the striatum of freely moving rats with an implanted glucose biosensor. J. Neurochem.

[61] Dai YQ, Shiu KK (2004). Highly sensitive amperometric glucose biosensor based on glassy carbon electrode with copper/palladium coating. Electroanalysis.

[62] Bartlett PN, Wang JH, James W (1998). Measurement of low glucose concentrations using a microelectrochemical enzyme transistor. Analyst.

[63] Wang J, Lu F (1998). Oxygen-rich oxidase enzyme electrodes for operation in oxygen-free solutions. J. Am. Chem. Soc.

[64] Wang J (1994). Selectivity coefficients for amperometric sensors. Talanta.

[65] Palmisano F, Rizzi R, Centonze D, Zambonin PG (2000). Simultaneous monitoring of glucose and lactate by an interference and cross-talk free dual electrode amperometric biosensor based on electropolymerized thin films. Biosens. Bioelectron.

[66] Ahmad F, Christenson A, Bainbridge M, Yusof APM, Ab Ghani S (2007). Minimizing tissue-material interaction in microsensor for subcutaneous glucose monitoring. Biosens. Bioelectron.

[67] Maalouf R, Chebib H, Saikali Y, Vittori O, Sigaud M, Garrelie F, Donnet C, Jaffirezic-Renault N (2007). Characterization of different diamond-like carbon electrodes for biosensor design. Talanta.

[68] Cooper JM, Pritchard DJ (1994). Biomolecular sensors for neurotransmitter determination electrochemical immobilization of glutamate oxidase at microelectrodes in a poly (o-phenylenediamine) film. J. Mater. Sci.: Mater. Electronl.

[69] Bao L, Avshalumov MV, Patel JC, Lee CR, Miller EW, Chang CJ, Rice ME (2009). Mitochondria are the source of hydrogen peroxide for dynamic brain-cell signaling. J. Neurosci.

[70] McMahon CP, Rocchitta G, Serra PA, Kirwan SM, Lowry JP, O’Neill RD (2006). The efficiency of immobilised glutamate oxidase decreases with surface enzyme loading: an electrostatic effect, and reversal by a polycation significantly enhances biosensor sensitivity. Analyst.

[71] Burmeister JJ, Palmer M, Gerhardt GA (2003). Ceramic-based multisite microelectrode array for rapid choline measures in brain tissue. Anal. Chim. Acta.

[72] Berners MOM, Boutelle MG, Fillenz M (1994). On-line measurement of brain glutamate with an enzyme/polymer-coated tubular electrode. Anal. Chem.

[73] Kulagina NV, Shankar L, Michael AC (1999). Monitoring glutamate and ascorbate in the extracellular space of brain tissue with electrochemical microsensors. Anal. Chem.

[74] Harrar JE (1963). Controlled-potential coulometric determination of hydrogen peroxide. Anal. Chem.

[75] Rothwell SA, McMahon CP, O’Neill RD (2010). Effects of polymerization potential on the permselectivity of poly(o-phenylenediamine) coatings deposited on Pt-Ir electrodes for biosensor applications. Electrochim. Acta.

[76] Hascup KN, Hascup ER, Pomerleau F, Huettl P, Gerhardt GA (2008). Second-by-second measures of L-glutamate in the prefrontal cortex and striatum of freely moving mice. J. Pharmacol. Exp. Ther.

[77] Morales-Villagran A, Medina-Ceja L, Lopez-Perez SJ (2008). Simultaneous glutamate and EEG activity measurements during seizures in rat hippocampal region with the use of an electrochemical biosensor. J. Neurosci. Meth.

[78] Du D, Ding JW, Cai J, Zhang AD (2007). One-step electrochemically deposited interface of chitosan-gold nanoparticles for acetylcholinesterase biosensor design. J. Electroanal. Chem.

[79] Dale N, Hatz S, Tian FM, Llaudet E (2005). Listening to the brain: microelectrode biosensors for neurochemicals. Trends Biotechnol.

[80] Patel BA, Arundell M, Parker KH, Yeoman MS, O’Hare D (2006). Detection of nitric oxide release from single neurons in the pond snail, Lymnaea stagnalis. Anal. Chem.

[81] Losito I, Palmisano F, Zambonin PG (2003). o-Phenylenediamine electropolymerization by cyclic voltammetry combined with electrospray ionization-ion trap mass spectrometry. Anal. Chem.

[82] Palmisano F, Zambonin PG, Centonze D (2000). Amperometric biosensors based on electrosynthesised polymeric films. Fresenius J. Anal. Chem.

[83] Centonze D, Losito I, Malitesta C, Palmisano F, Zambonin PG (1997). Electrochemical immobilisation of enzymes on conducting organic salt electrodes: characterisation of an oxygen independent and interference-free glucose biosensor. J. Electroanal. Chem.

[84] Wang Q, Tang H, Xie QJ, Jia XE, Zhang YY, Tan L, Yao SZ (2008). The preparation and characterization of poly(o-phenylenediamine)/gold nanoparticles interface for immunoassay by surface plasmon resonance and electrochemistry. Colloid. Surface. B.

[85] Camurri G, Ferrarini P, Giovanardi R, Benassi R, Fontanesi C (2005). Modelling of the initial stages of the electropolymerization mechanism of o-phenylenediamine. J. Electroanal. Chem.

[86] Losito I, De Giglio E, Cioffi N, Malitesta C (2001). Spectroscopic investigation on polymer films obtained by oxidation of o-phenylenediamine on platinum electrodes at different pHs. J. Mater. Chem.

[87] Ekinci E, Erdogdu G, Karagozler AE (2001). Preparation, optimization, and voltammetric characteristics of poly(o-phenylenediamine) film as a dopamine-selective polymeric membrane. J. Appl. Polym. Sci.

[88] Centonze D, Malitesta C, Palmisano F, Zambonin PG (1994). Permeation of solutes through an electropolymerized ultrathin poly-o-phenylenediamine film used as an enzyme-entrapping membrane. Electroanalysis.

